# Associations of the cerebrospinal fluid hepatocyte growth factor with Alzheimer’s disease pathology and cognitive function

**DOI:** 10.1186/s12883-021-02356-9

**Published:** 2021-10-06

**Authors:** Li-Jing Zhao, Zuo-Teng Wang, Ya-Hui Ma, Wei Zhang, Qiang Dong, Jin-Tai Yu, Lan Tan, Alzheimer’s Disease Neuroimaging Initiative

**Affiliations:** 1 Department of Neurology, Qingdao Municipal Hospital, Dalian Medical University, Dalian, China; 2 College of Medicine and Pharmaceutics, Ocean University of China, Qingdao, China; 3 Department of Neurology, Qingdao Municipal Hospital, Qingdao University, Qingdao, China; 4 Department of Neurology and Institute of Neurology, Huashan Hospital, Shanghai Medical College, Fudan University, 12th Wulumuqi Zhong Road, Shanghai, 200040 China

**Keywords:** Alzheimer’s disease, Hepatocyte growth factor, Cerebrospinal fluid, Cognition

## Abstract

**Background:**

Hepatocyte growth factor (HGF) plays a role in neuronal survival and development, and has been implicated in neurodegenerative diseases. We sought to examine the associations of the CSF HGF with Alzheimer’s disease (AD) pathology and cognitive function.

**Methods:**

A total of 238 participants (including 90 cognitively normal (CN) and 148 mild cognitive impairment (MCI)) who had measurements of CSF HGF were included from the Alzheimer’s Disease Neuroimaging Initiative (ADNI) database. Multiple linear regression models were utilized to explore the cross-sectional associations of CSF HGF with AD biomarkers (including Aβ42, pTau, and tTau proteins) in non-demented participants. Moreover, linear mixed-effects regression models were utilized to explore the longitudinal associations of HGF subgroups with cognitive function. Mediation analyses were utilized to explore the mediation effects of AD markers.

**Results:**

MCI individuals had significantly increased CSF HGF compared with the CN individuals. Results of multiple linear regressions showed significant correlations of CSF HGF with CSF Aβ42, pTau, and tTau in non-demented participants. Higher level of baseline CSF HGF was associated with faster cognitive decline. Influences of the baseline CSF HGF on cognition were partially mediated by Aβ42, pTau, and tTau pathologies.

**Conclusions:**

High concentrations of HGF in CSF may be related to faster cognitive decline. The cognitive consequences of higher CSF HGF partly stem from AD pathology, which suggests that the CSF HGF may be an attractive biomarker candidate to track AD progression.

**Supplementary Information:**

The online version contains supplementary material available at 10.1186/s12883-021-02356-9.

## Background

Alzheimer’s disease (AD) is pathologically characterized by aggregated amyloid plaques and neurofibrillary tangles (NFTs) [1]. Amyloid-β (Aβ) and tau/phosphorylated tau (pTau) biomarkers in cerebrospinal fluid (CSF) have been identified as reliable diagnostic biomarkers of AD pathology [2, 3]. Hepatocyte growth factor (HGF) is a plasminogen-like protein consisting of light and heavy chains of 35 and 65 kDa. The receptor of HGF was identified as the c-Met protooncogene product of transmembrane receptor tyrosine kinase (RTK) [4, 5]. HGF-cMet system is involved in a wide range of cellular targets (including epithelial, endothelial, and neurons et al.) [6]. HGF-induced signaling through the receptor Met initiates a series of cellular responses (including mitogenesis, cell motility and morphogenesis et al.) [6, 7]. It has been reported that the HGF can express in the mammalian central nervous system (CNS) [8–11]. HGF immunoreactivity was present in astrocytes and microglia, which was increased in both lacunar strokes and AD [11, 12]. HGF-Met system may plays an important role in microglial reactions to CNS injuries [13]. Moreover, several studies suggested that the HGF levels were increased in brain tissue, cerebrospinal fluid (CSF), and serum of AD patients [12, 14, 15]. At present, there is insufficient evidence on associations between HGF and AD pathology. Here, the aim of this study was to evaluate the association of CSF HGF with CSF AD biomarkers and cognitive function. We also sought to explore whether the AD pathology mediated the associations between the HGF and cognitive function.

## Methods

### ADNI database

Alzheimer’s Disease Neuroimaging Initiative (ADNI) data are deposited in a open access repository and can be accessed at http://adni.loni.usc.edu. The ADNI (launched in 2003) was led by Dr. Michael W. Weiner. All participants were recruited from more than 50 sites across the Canada and United State. The ADNI aim to develop clinical, biochemical, and imaging biomarkers for tracking AD. ADNI was approved by all regional ethical committees, and contained written informed-consent documents of all participants.

### Participants

From ADNI, we included only cognitively normal (CN) (*n* = 90), early MCI or late MCI (*n* = 148) participants. Cerebral amyloid-β accumulation generally precedes the dementia stage by many years. We excluded individuals with dementia (due to AD), since the aim of this study was to examine detection of the earliest accumulation of amyloid-β. All participants underwent assessments of CSF HGF and CSF AD biomarkers at baseline. All participants were adults aged 55 to 90 years. The specific ADNI diagnostic criteria for distinguishing CN, MCI, and AD participants were summarized in Additional Table 1 [16, 17]. CN and MCI subjects were defined as non-demented individuals in the study.

### CSF measurements

In ADNI, CSF procedural protocols have been described previously. INNOBIA AlzBio3 immunoassay (Fujirebio, Belgium) was used to measure CSF AD biomarkers (including CSF Aβ_1–42_, total tau (tTau), and pTau (pg/ml)). The within-batch precision values were < 10% (5.1–7.8% for Aβ_1–42_, 4.4–9.8% for tTau and 5.1–8.8% for pTau).

CSF HGF was measured using a multiplex panel. The multiplex panel is based upon Luminex immunoassay technology and has been developed by Rules Based Medicine (MyriadRBM) to measure a range of inflammatory, metabolic, lipid and other disease relevant indices. Quality control (QC) data that is specific for the CSF samples included in this study are the test/retest results for the 16 randomly selected CSF samples (http://adni.loni.usc.edu). Analytes were removed if the mean percentage difference was greater than 35% or the test-retest sample was less than 7 or the mean absolute percentage difference was greater than 60% or if the Bland-Altman slope and intercept significantly differed from zero. Participants who had extreme outliers (< 3-fold or > 3-fold standard deviations (SD) from the mean value) were removed. Finally, 5 participants were removed from the data set.

### Cognitive assessment

Three cognitive measures (Mini-Mental State Examination (MMSE), ADNI memory (ADNI-MEM), and ADNI executive function (ADNI-EF)) were used to evaluating cognitive functions in this study. The ADNI-MEM was developed from the Logical Memory Test, Rey Auditory Verbal Learning Test, MMSE, and Alzheimer’s Disease Assessment Scale cognitive subscale (ADAS-Cog) [18]. ADNI-EF consists of Category Fluency-vegetables, 5 Clock Drawing items (circle, symbol, numbers, hands, time), Category Fluency-animals, Digit Span Backwards, Wechsler Adult Intelligence Scale-Revised (WAIS-R) Digit Symbol Substitution, and Trail-Making Test parts (A and B) [19].

### APOE ε4 genotyping

The APOE genotyping was performed by Hhal restriction enzyme digestion, polymerase chain reaction (PCR) amplification, and standard gel resolution and visualization processes [20, 21]. Quality-controlled genotyping data were obtained from the database. Individuals were classified as carriers of one *APOE ε4* allele, carriers of two *APOE ε4* alleles, and *APOE ε4* non-carriers.

### White matter hyperintensities (WMHs) measurement

All subjects were examined using 3.0-Tesla MRI scanner. The procedure is described in previous studies [22, 23]. All parameters were available through the database website (http://adni.loni.usc.edu/methods/mri-tool/mri-analysis). White matter hyperintensities volume (WMHV) were measured using the Bayesian approach for the segmentation of high-resolution 3D MPRAGE T1-weighted and T2-FLAIR sequences [24].

### Statistical analysis

We used ANOVA, non-parametric Kruskal-Wallis H test, and Chi-squared test to compare the baseline clinical and demographic characteristics. In addition, pearson correlation tests (for continuous variables) were used to explore the associations between the baseline CSF AD biomarkers (normally distributed) and CSF HGF. All the non-demented participants were grouped by tertiles of CSF HGF at baseline (Group A lowest tertile; Group B middle tertile; Group C highest tertile). We used multiple linear regressions to explore the associations of HGF subgroups (independent variable) with CSF AD biomarkers and cognition (dependent variables) (covariates including age, sex, years of education, *APOE4* status, and diagnosis). We used Tukey HSD post hoc test to perform pairwise multiple comparisons. We used linear mixed-effects regression models to explore the longitudinal relationship of HGF subgroups with cognitive function (covariates including age, sex, years of education, *APOE4* status, baseline diagnosis, and baseline cognitive status). GraphPad Prism 8.00 and R version 3.6.2 software were used for statistical analyses and figure preparation.

Longitudinal rates of change in cognitive function (including MMSE, ADNI-MEM, and ADNI-EF) were computed by using linear mixed models (covariates including age, sex, years of education, *APOE4* status, diagnosis, and baseline cognitive status). We estimated the mean rates of change (by the sim function in the arm package with 10,000 replicates) for the whole samples [25]. Mediation analyses were performed to test and quantify the mediation effects of AD pathology on the associations of the CSF HGF with cognitive function (covariates including age, sex, education, and *APOE4* status). We used bootstrapping (10,000 iterations) methods to estimate the 95% CI [26]. These analyses were performed by using R software packages (“lm”, “arm”, “lme4”, “ggplot2”, and “mediation”). *P* < 0.05 was considered significant.

## Results

### Characteristics of participants

**Fig. 1:**
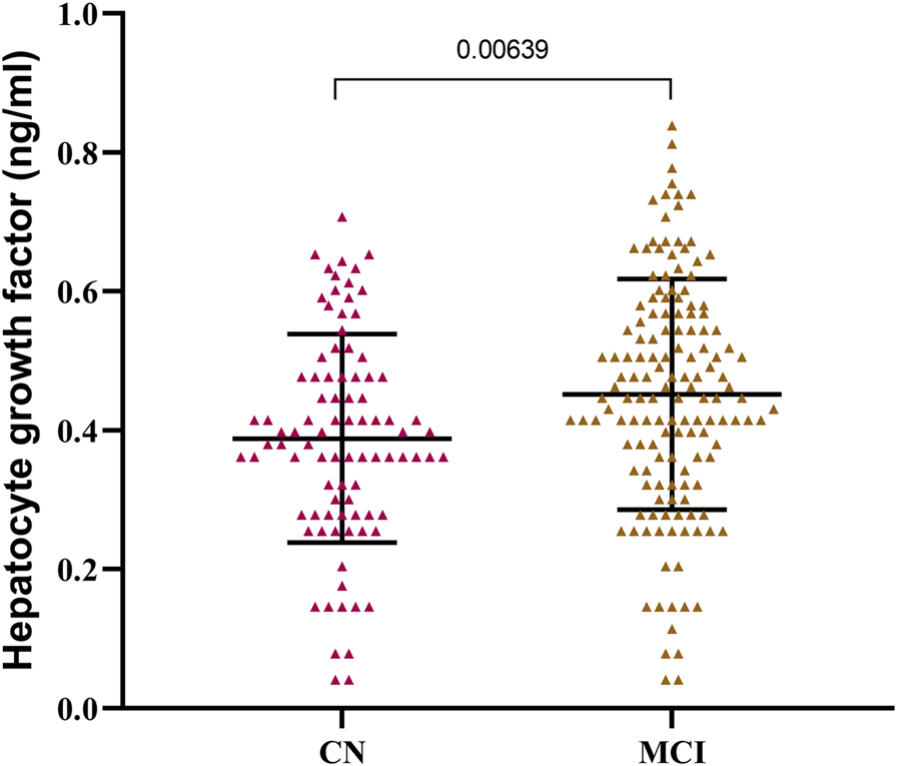
Associations of diagnosis with baseline CSF HGF. CSF HGF was significantly higher in MCI (P = 0.00639) compared to CN participants

A total of 238 participants (including 90 CN and 148 MCI) who had measurements of CSF AD biomarkers (including Aβ42, pTau, and tTau proteins) and HGF were included. Demographical and clinical characteristics are described in Table 1. The participants were aged 56 to 90 (mean ± SD age, 75.1 ± 6.6) years. The study population had a female proportion of 40.3%, 15.8 ± 2.9 years of education, and an *APOE4* positive percentage of 42.4%. The level of CSF HGF was significantly higher in MCI (*P* = 0.00639) compared to CN participants (Figure 1and Table 1). There was no significant difference between the diagnosis subgroups in WMHV (*P* = 0.894) (Table 1).

**Table 1:** Demographic characteristics for all the participants

Characteristics	CN	MCI	P
N	90	148	
Age (Mean ± SD, year)	75.60 ± 5.46	74.82 ± 7.21	0.353
Sex (Female, %)	45 (50)	47 (31.8)	0.005
Education (Mean ± SD, year)	15.57 ± 2.94	15.96 ± 2.94	0.284
*APOE* ε4 carrier status (Yes, %)	22 (24.4)	79 (53.4)	< 0.0001
WMHV (Mean ± SD, cm^3^)	0.77 ± 1.93	0.82 ± 2.64	0.894
CSF HGF (Mean ± SD, ng/ml)	0.39 ± 0.15	0.45 ± 0.17	0.006

ANOVA, non-parametric Kruskal-Wallis H test, and Chi-squared test were used to compare the baseline demographic and clinical characteristics. Abbreviations: *CN* cognitively normal; *MCI* mild cognitive impairment; *WMHV* white matter hyperintensities volume; *CSF* cerebrospinal fluid; *HGF* Hepatocyte Growth Factor

**Fig. 2:**
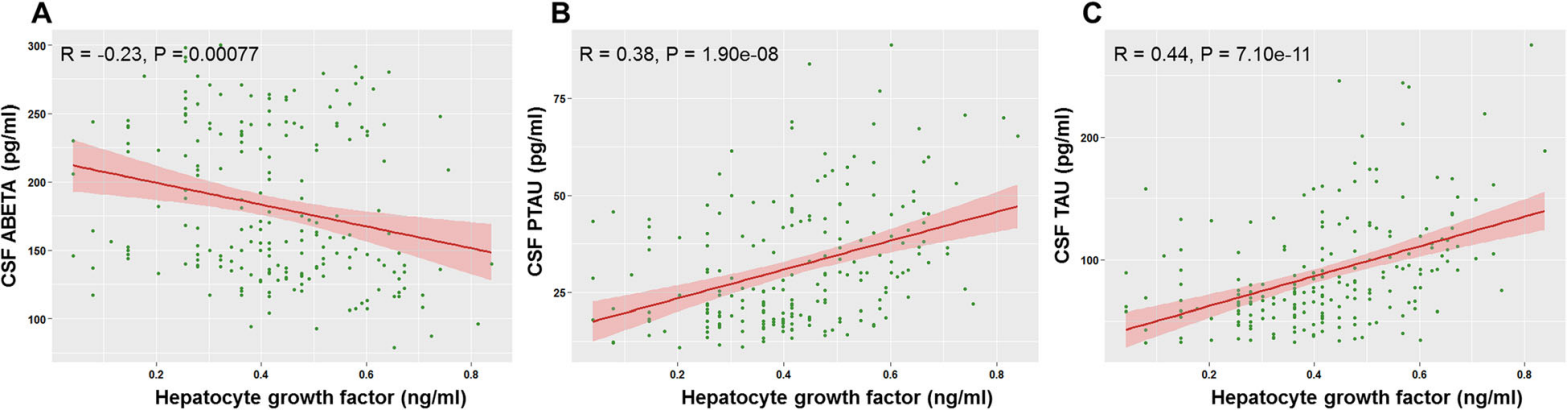
Associations of baseline CSF HGF with CSF AD biomarkers in non-demented participants. The baseline CSF HGF is significant associated with the CSF Aβ42 (*R* = − 0.23, P = 0.00077) (**A**), CSF pTau (R = 0.38, P = 1.90e-08) (**B**), and CSF tTau (R = 0.44, *P* = 7.10e-11) (**C**)

**Fig. 3:**
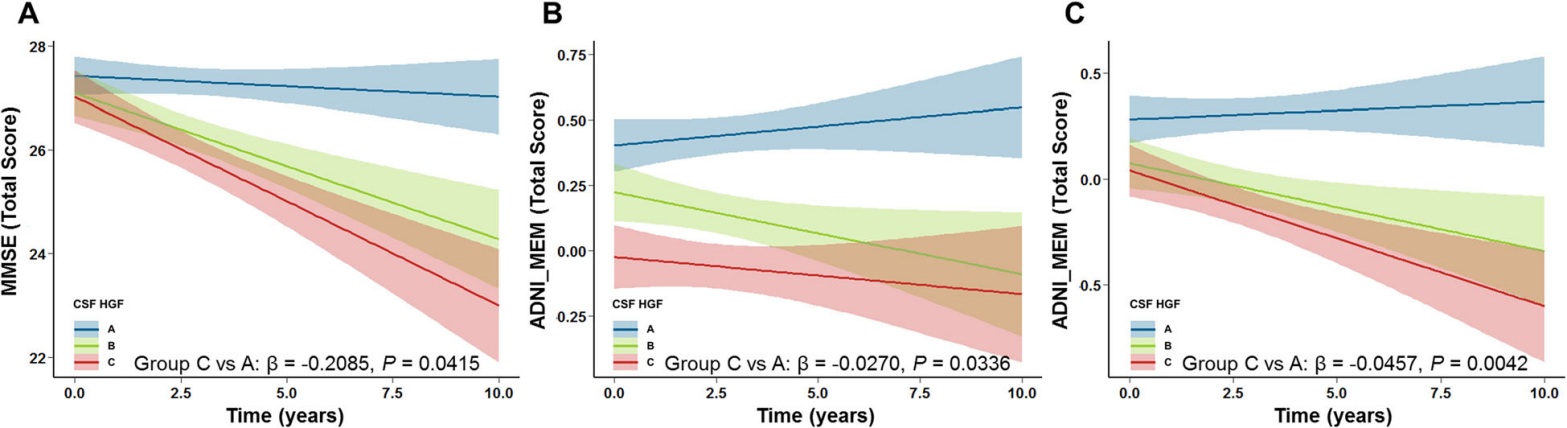
Associations of the CSF HGF with longitudinal changes in cognitive function in non-demented participants. Longitudinal rates of change in cognitive function (including MMSE (**A**), ADNI-MEM (**B**), and ADNI-EF (**C**)) were computed by using linear mixed models. All models were adjusted for age, sex, years of education, APOE4 status, baseline diagnosis, and baseline cognitive status

The baseline CSF HGF is significant associated with the CSF Aβ42 (R = − 0.23, *P* = 0.00077), CSF pTau (R = 0.38, *P* = 1.90e-08), and CSF tTau (R = 0.44, *P* = 7.10e-11) in non-demented participants (Fig. 2). The associations between CSF HGF and CSF AD biomarkers (stratified by diagnosis) are presented in Additional Figure 1 to Figure 3. The demographic and clinical characteristics of non-demented participants (stratified by HGF concentration) are presented in Table 2. Baseline demographics were similar in the three subgroups (except mean levels of age). There was no significant difference in WMHV between different diagnostic groups and CSF HGF subgroups (Table 1 and Table 2). After controlling for a range of potential confounders (age, sex, years of education, *APOE4* status, and diagnosis), the HGF tertiles were significantly associated with baseline CSF Aβ42 (β = − 8.4660, *p* = 0.0358), pTau (β = 6.2368, *p* = 3.96e-07), and tTau (β = 21.6103, *p* = 3.00e-10) (Table 3). Significant associations were not found between the CSF HGF and baseline cognitive status (Table 3). Using post hoc tests (Tukey HSD), it was found that CSF Aβ42 was reduced among Group C participants compared to Group A participants. CSF pTau and CSF tTau were increased among Group C participants, as compared to Group A and Group B participants. (Additional Table 2).

**Table 2:** Demographic characteristics for the non-demented participants

Characteristics	Group A	Group B	Group C	P
N	84	85	69	
Age (Mean ± SD, year)	74.52 ± 6.65	73.92 ± 6.18	77.30 ± 6.60	0.0038
Sex (Female, %)	39 (46.4)	30 (35.3)	23 (33.3)	0.1872
Education (Mean ± SD, year)	15.73 ± 2.94	15.67 ± 3.13	16.08 ± 2.71	0.6551
*APOE* ε4 carrier status (Yes, %)	33 (39.3)	33 (38.8)	35 (50.7)	0.5560
WMHV (Mean ± SD, cm^3^)	1.03 ± 3.49	0.52 ± 0.92	0.87 ± 1.98	0.076
CSF HGF (Mean ± SD, ng/ml)	0.25 ± 0.09	0.44 ± 0.04	0.62 ± 0.07	< 0.0001

All participants were grouped by tertiles of CSF HGF at baseline. Group A lowest tertile; Group B middle tertile; Group C highest tertile. ANOVA, non-parametric Kruskal-Wallis H test, and Chi-squared test were used to compare the baseline demographic and clinical characteristics. Abbreviations: *WMHV* white matter hyperintensities volume; *CSF* cerebrospinal fluid; *HGF* Hepatocyte Growth Factor

**Table 3:** The associations of CSF HGF groups with baseline CSF AD biomarkers and cognitive function

Dependent variable	CSF HGF	
	beta	***p*** value
CSF Aβ42	−8.4660	**0.0358**
CSF pTau	6.2368	**3.96e-07**
CSF tTau	21.6103	**3.00e-10**
MMSE scores	0.0449	0.7268
ADNI-MEM scores	−0.0461	0.3075
ADNI-EF scores	−0.0661	0.2953

Multiple linear regression models were utilized to explore these associations. All models were adjusted for age, sex, years of education, *APOE4* status, and diagnosis at baseline. Group A lowest tertile; Group B middle tertile; Group C highest tertile. Abbreviations: *AD* Alzheimer’s disease; *CSF* cerebrospinal fluid; *HGF* Hepatocyte Growth Factor

### Longitudinal relationship between CSF HGF and cognitive function

After controlling for a range of potential confounders (age, sex, years of education, APOE4 status, baseline diagnosis, and baseline cognitive status), individuals in group C (the highest tertile) showed faster decline in MMSE (β = − 0.2155, *P* = 0.0371), ADNI_MEM (β = − 0.0271, *P* = 0.0397), and ADNI_EF (β = − 0.0442, *P* = 0.0037) compared to group A (the lowest tertile) (Fig. 3 and Additional Table 3).

### Causal mediation analyses

**Fig. 4:**
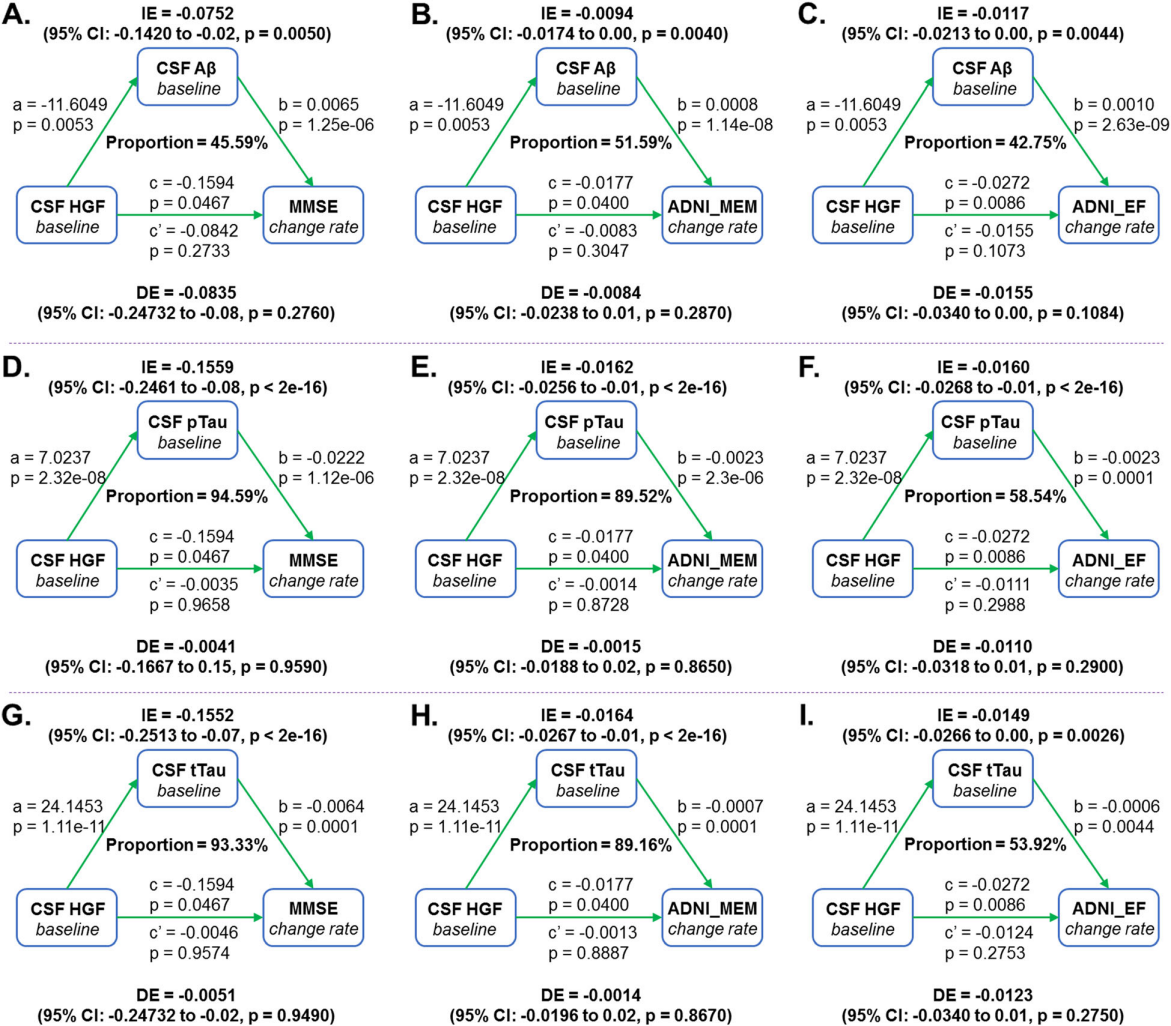
Mediation effects of baseline AD pathology on the associations of CSF HGF with cognitive function in non-demented participants. Results of mediation analyses suggested that 45.59% of the total association of the CSF HGF with the decline in MMSE scores (**A**), 51.59% of the total association of the CSF HGF with the decline in ADNI-MEM scores (**B**), and 42.75% of the total association with the decline in ADNI-EF scores (C), were attributed to baseline Aβ42. Moreover, 94.59% of the total association of the CSF HGF with the decline in MMSE scores (D), 89.52% of the total association of the CSF HGF with the decline in ADNI-MEM scores (E), and 58.54% of the total association with the decline in ADNI-EF scores (F), were attributed to baseline pTau. In addition, 93.33% of the total association of the CSF HGF with the decline in MMSE scores (G), 89.16% of the total association of the CSF HGF with the decline in ADNI-MEM scores (H), and 53.92% of the total association with the decline in ADNI-EF scores (I), were attributed to baseline tTau. All models were adjusted for age, sex, years of education, and *APOE4* status

We investigated whether these AD pathology mediated the influences of CSF HGF on cognitive function. After controlling for age, sex, years of education, and *APOE4* status, 45.59% of the total association of the CSF HGF with the decline in MMSE scores (Fig. 4A), 51.59% of the total association of the CSF HGF with the decline in ADNI-MEM scores (Fig. 4B), and 42.75% of the total association with the decline in ADNI-EF scores (Fig. 4C), were attributed to baseline Aβ42. Moreover, 94.59% of the total association of the CSF HGF with the decline in MMSE scores (Fig. 4D), 89.52% of the total association of the CSF HGF with the decline in ADNI-MEM scores (Fig. 4E), and 58.54% of the total association with the decline in ADNI-EF scores (Fig. 4E), were attributed to baseline pTau. In addition, 93.33% of the total association of the CSF HGF with the decline in MMSE scores (Fig. 4G), 89.16% of the total association of the CSF HGF with the decline in ADNI-MEM scores (Fig. 4H), and 53.92% of the total association with the decline in ADNI-EF scores (Fig. 4I), were attributed to baseline tTau.

## Discussion

To our knowledge, this is the first study to explore the association between CSF HGF and AD biomarkers. MCI participants had higher levels of CSF HGF compared to CN participants. Higher concentration of CSF HGF was related to higher levels of CSF markers of AD pathology and faster cognitive decline in non-demented participants. Influences of CSF HGF on cognition were partially mediated by AD pathology.

It has been reported that the HGF level was increased in CSF in AD, which was in line with our findings [14]. Previous studies have confirmed that HGF is expressed in various brain cell types (including neurons, astrocytes, microglia, and oligodendrocytes) [11, 12, 27]. Astrocytes involved in regeneration of neurons and glia may played important roles in HGF production in CSF [28]. Microglia- and astroglia- mediated innate immune responses are associated with aggregated and misfolded proteins, which contribute to clinical staging and disease progression in AD [29, 30]. The HGF immunoreactivity was presented in neurons, microglia and astrocytes, which was increased in AD [11, 12]. This increase in HGF immunoreactivity is most likely due to the proliferation of both reactive microglia and astrocytes around the periphery of senile plaques. The HGF may be expressed in reaction to AD pathology [12]. In addition, the production of HGF in the CSF might reflect damage and repair of white matter of the brain and spinal cord in neurologic disease [28]. Tsuboi et al. found that the concentration of CSF HGF could reflect white matter damage in AD individuals [14]. There were direct links between AD pathology and white matter macrostructural and microstructural damage, which suggested a potential correlation between HGF and AD pathology [31, 32].

The cMet is a multifunctional protein receptor that binds HGF and able to mediate all the known effects of HGF [33]. HGF-cMet signaling induces cell proliferation, glucose metabolism, neuroregeneration, and neuroprotection [34–36]. Hamasaki et al.’ study indicated that the cMet level was decreased in granule cells of the dentate gyrus and hippocampal pyramidal neurons in AD brains [37]. In particular, cMet in hippocampal pyramidal neurons decreased more significantly than in neocortical neurons [37]. It has reported that the HGF partially mediates cognitive enhancement, as well as structural and functional recovery by activating cMet-AKT-GSK3β signaling pathway in the AD hippocampus [38]. Moreover, HGF can mediate tau hyperphosphorylation via activation of cMet-AKT-GSK3β signaling pathway [38]. The cMET plays a central role in neurotrophic, and its decline may adversely affect neuronal survival. HGF maybe increase reactively in the AD brains. Because the hippocampus play important roles in cognitive function, the decrease in cMet level may help to explain, at least in part, the relationships between CSF HGF and cognitive function [37]. In addition, higher levels of serum HGF were also associated with pre-dementia and AD [15]. HGF is relatively stable in serum, and can crosses the blood-brain barrier (BBB) in an intact form [39]. At least a third of HGF can reach the cerebral circulation [38]. The serum HGF may be a potential biomarker for small vessel disease in MCI and AD individuals. In addition, the correlation between AD pathology and BBB dysfunction has been well-reported [40, 41]. A vicious circle between neurovascular unit impairments and Aβ accumulation may exist during disease progression [40]. The increase of CSF HGF may also be partially caused by increased permeability via BBB. The BBB may play a potential mediational role in the relationships between CSF HGF and cognition. Moreover, hypoperfusion and BBB impairment appear in the normal-appearing white matter (NAWM) and WMH, which increase in the proximity of the WMH [42]. More sensitive indicators may be needed to measure BBB functionality. The more detailed mechanisms need to be explored further.

There are limitations in this study. First, we did not control for other potential confounding factors (e.g., other genetic factors, vascular risk factors, and socioeconomic status et al.) in our analyses. Second, we did not discuss the association between the longitudinal changes of CSF HGF and AD pathology due to the limited data. Third, this study was conducted with modest samples sizes, and the generalizability of our conclusions might be restricted by sources of studied populations of ADNI which recruited participants from volunteers. Further large-scale community-based longitudinal studies are warranted to validate these associations.

## Conclusions

The present study showed that the CSF HGF levels were significantly increased in MCI individuals. High concentrations of HGF in CSF may be related to AD pathology and faster cognitive decline in non-demented participants. AD pathology may act as a key mediator for influences of HGF on cognitive function. The CSF HGF may be an attractive biomarker candidate to track AD progression.

## Supplementary Information

**Additional file 1: Table 1**. Specific components of ADNI classification criteria to distinguish CN, Early MCI, Late MCI, and AD. The specific ADNI diagnostic criteria for distinguishing CN, MCI, and AD participants were summarized in Additional Table 1. **Table 2.** Tukey post hoc test for multiple comparisons between CSF HGF groups and CSF AD biomarkers and cognitive function. Using post hoc tests (Tukey HSD), it was found that CSF Aβ42 was reduced among Group C participants compared to Group A participants. CSF pTau and CSF tTau were increased among Group C participants, as compared to Group A and Group B participants. **Table 3.** Multiple comparisons between CSF HGF and longitudinal changes in cognitive function. After controlling for a range of potential confounders (age, sex, years of education, APOE4 status, baseline diagnosis, and baseline cognitive status), individuals in group C (the highest tertile) showed faster decline in MMSE (β = − 0.2155, *P* = 0.0371), ADNI_MEM (β = − 0.0271, *P* = 0.0397), and ADNI_EF (β = − 0.0442, *P* = 0.0037) compared to group A (the lowest tertile). **Figure 1.** Associations of baseline CSF HGF with CSF AD biomarkers in non-demented participants (stratified by diagnosis). The baseline CSF HGF is significant associated with the CSF Aβ42 in MCI (R = − 0.19, *P* = 0.045), but not in CN (R = − 0.17, *P* = 0.12). **Figure 2.** Associations of baseline CSF HGF with CSF AD biomarkers in non-demented participants (stratified by diagnosis). The baseline CSF HGF is significant associated with the CSF pTau (CN, R = 0.35, *P* = 8E-04; MCI, R = 0.34, *P* = 2E-04). **Figure 3.** Associations of baseline CSF HGF with CSF AD biomarkers in non-demented participants (stratified by diagnosis). The baseline CSF HGF is significant associated with the CSF tTau (CN, R = 0.44, *P* = 1.6E-05; MCI, R = 0.41, *P* = 6.8E-05).

## Data Availability

The datasets used and/or analyzed during the current study are available from the corresponding author on reasonable request.

## Abbreviations

Aβ: Amyloid-beta
AD: Alzheimer’s disease
ADAS-Cog: Alzheimer’s disease assessment scale cognitive subscale
ADNI: Alzheimer’s disease neuroimaging initiative
APOE: Apolipoprotein E
BBB: Blood-brain barrier
CN: Cognitively normal
CNS: Central nervous system
CSF: Cerebrospinal fluid
HGF: Hepatocyte growth factor
MCI: mild cognitive impairment
MMSE: Mini-mental state examination
PCR: Polymerase chain reaction
pTau: Phosphorylated tau
QC: Quality control
RTK: Receptor tyrosine kinase
SD: Standard deviations
tTau: Total tau
WAIS-R: Wechsler adult intelligence scale-revised
