# Supplementary material for: Associations of the cerebrospinal fluid hepatocyte growth factor with Alzheimer’s disease pathology and cognitive function
Source: BMC Neurol. 2021 Oct 6;21:387. doi: 10.1186/s12883-021-02356-9 (PMC8493684; doi:10.1186/s12883-021-02356-9)
Supplement: Supplementary file 1 — Additional file 1: Table 1. Specific components of ADNI classification criteria to distinguish CN, Early MCI, Late MCI, and AD. The specific ADNI diagnostic criteria for distinguishing CN, MCI, and AD participants were summarized in Additional Table 1. Table 2. Tukey post hoc test for multiple comparisons between CSF HGF groups and CSF AD biomarkers and cognitive function. Using post hoc tests (Tukey HSD), it was found that CSF Aβ42 was reduced among Group C participants compared to Group A participants. CSF pTau and CSF tTau were increased among Group C participants, as compared to Group A and Group B participants. Table 3. Multiple comparisons between CSF HGF and longitudinal changes in cognitive function. After controlling for a range of potential confounders (age, sex, years of education, APOE4 status, baseline diagnosis, and baseline cognitive status), individuals in group C (the highest tertile) showed faster decline in MMSE (β = − 0.2155, P = 0.0371), ADNI_MEM (β = − 0.0271, P = 0.0397), and ADNI_EF (β = − 0.0442, P = 0.0037) compared to group A (the lowest tertile). Figure 1. Associations of baseline CSF HGF with CSF AD biomarkers in non-demented participants (stratified by diagnosis). The baseline CSF HGF is significant associated with the CSF Aβ42 in MCI (R = − 0.19, P = 0.045), but not in CN (R = − 0.17, P = 0.12). Figure 2. Associations of baseline CSF HGF with CSF AD biomarkers in non-demented participants (stratified by diagnosis). The baseline CSF HGF is significant associated with the CSF pTau (CN, R = 0.35, P = 8E-04; MCI, R = 0.34, P = 2E-04). Figure 3. Associations of baseline CSF HGF with CSF AD biomarkers in non-demented participants (stratified by diagnosis). The baseline CSF HGF is significant associated with the CSF tTau (CN, R = 0.44, P = 1.6E-05; MCI, R = 0.41, P = 6.8E-05). [file 12883_2021_2356_MOESM1_ESM.docx]

**Additional Table 1.**

**Specific components of ADNI classification criteria to distinguish CN, Early MCI, Late MCI, and AD.**

|  | **CN** | **Early MCI** | **Late MCI** | **AD** |
| --- | --- | --- | --- | --- |
| Subjective complaint | None, aside from those common to other normal subjects of that age range. | Yes, by subject (verified by study partner) or study partner or clinician | Yes, by subject (verified by study partner) or study partner or clinician | Yes, by subject (verified by study partner) or study partner or clinician |
| MMSE | ≥24 | ≥24 | ≥24 | 20-26 (Inclusive) |
| Logical Memory | ≥9 for 16+ years of education | 9-11 for 16+ years of education | ≤8 for 16+ years of education | ≤8 for 16+ years of education |
|  | ≥5 for 8-15 years of education | 5-9 for 8-15 years of education | ≤4 for 8-15 years of education | ≤4 for 8-15 years of education |
|  | ≥3 for 0-7 years of Education | 3-6 for 0-7 years of education | ≤2 for 0-7 years of education | ≤2 for 0-7 years of education |
| CDR | CDR=0 | CDR=0.5 | CDR=0.5 | CDR=0.5 or 1.0 |
|  | Memory Box score must be 0 | Memory Box score of at least 0.5 | Memory Box score of at least 0.5 |  |
| General cognition and functional status | Cognitively normal, based on an absence of significant impairment in cognitive functions or activities of daily living. | General cognition and functional performance sufficiently preserved such that a diagnosis of AD cannot be made. | General cognition and functional performance sufficiently preserved such that a diagnosis of AD cannot be made. | NINCDS/ADRDA criteria for probable AD |
| Abbreviations: CN=Cognitively Normal; MCI=Mild Cognitive Impairment; AD=Alzheimer’s Disease; MMSE=Mini Mental State Exam; CDR=Clinical Dementia Rating; NINCDS/ADRDA=National Institute of Neurological and Communication Disorders and Stroke/Alzheimer’s Disease and Related Disorders Association. Late MCI criteria were the only MCI criteria for ADNI-1; Early MCI criteria were only included in ADNI-GO and ADNI-2. (Petersen et al., 2010; Thomas et al., 2019) | | | | |

**Additional Table 2.**

**Tukey post hoc test for** **multiple comparisons between CSF HGF groups and CSF AD biomarkers and cognitive function**

| **Dependent variable** | **Group I** | **Group J** | **Mean Difference (I-J)** | **Std. Error** | **Sig.** | **95% CI** | |
| --- | --- | --- | --- | --- | --- | --- | --- |
|  |  |  |  |  |  | **Lower Bound** | **Upper Bound** |
| CSF Aβ42 | Group A | Group B | 27.2749 | 8.9560 | **0.0070** | 6.1290 | 48.4210 |
|  |  | Group C | 27.4758 | 9.4288 | **0.0110** | 5.2130 | 49.7380 |
|  | Group B | Group A | -27.2749 | 8.9560 | **0.0070** | -48.4210 | -6.1290 |
|  |  | Group C | 0.2008 | 9.6292 | 1.0000 | -22.5350 | 22.9370 |
|  | Group C | Group A | -27.4758 | 9.4288 | **0.0110** | -49.7380 | -5.2130 |
|  |  | Group B | -0.2008 | 9.6292 | 1.0000 | -22.9370 | 22.5350 |
| CSF pTau | Group A | Group B | -5.9187 | 2.4353 | **0.0420** | -11.6687 | -0.1687 |
|  |  | Group C | -15.0790 | 2.5639 | **﹤0.0001** | -21.1326 | -9.0254 |
|  | Group B | Group A | 5.9187 | 2.4353 | **0.0420** | 0.1687 | 11.6687 |
|  |  | Group C | -9.1603 | 2.6184 | **0.0020** | -15.3426 | -2.9780 |
|  | Group C | Group A | 15.0790 | 2.5639 | **﹤0.0001** | 9.0254 | 21.1326 |
|  |  | Group B | 9.1603 | 2.6184 | **0.0020** | 2.9780 | 15.3426 |
| CSF tTau | Group A | Group B | -21.0236 | 6.7595 | **0.0060** | -36.9837 | -5.0636 |
|  |  | Group C | -49.1232 | 7.1164 | **﹤0.0001** | -65.9259 | -32.3206 |
|  | Group B | Group A | 21.0236 | 6.7595 | **0.0060** | 5.0636 | 36.9837 |
|  |  | Group C | -28.0996 | 7.2677 | **﹤0.0001** | -45.2594 | -10.9398 |
|  | Group C | Group A | 49.1232 | 7.1164 | **﹤0.0001** | 32.3206 | 65.9259 |
|  |  | Group B | 28.0996 | 7.2677 | **﹤0.0001** | 10.9398 | 45.2594 |
| MMSE  scores | Group A | Group B | 0.3410 | 0.2870 | 0.4610 | -0.3400 | 1.0200 |
|  |  | Group C | 0.4930 | 0.3030 | 0.2370 | -0.2200 | 1.2100 |
|  | Group B | Group A | -0.3410 | 0.2870 | 0.4610 | -1.0200 | 0.3400 |
|  |  | Group C | 0.1520 | 0.3020 | 0.8710 | -0.5600 | 0.8600 |
|  | Group C | Group A | -0.4930 | 0.3030 | 0.2370 | -1.2100 | 0.2200 |
|  |  | Group B | -0.1520 | 0.3020 | 0.8710 | -0.8600 | 0.5600 |
| ADNI-MEM scores | Group A | Group B | 0.1883 | 0.1189 | 0.2540 | -0.0920 | 0.4687 |
|  |  | Group C | 0.3756 | 0.1255 | **0.0090** | 0.0795 | 0.6716 |
|  | Group B | Group A | -0.1883 | 0.1189 | 0.2540 | -0.4687 | 0.0920 |
|  |  | Group C | 0.1873 | 0.1252 | 0.2950 | -0.1080 | 0.4825 |
|  | Group C | Group A | -0.3756 | 0.1255 | **0.0090** | -0.6716 | -0.0795 |
|  |  | Group B | -0.1873 | 0.1252 | 0.2950 | -0.4825 | 0.1080 |
| ADNI-EF  scores | Group A | Group B | 0.2330 | 0.1282 | 0.1660 | -0.0693 | 0.5353 |
|  |  | Group C | 0.3488 | 0.1353 | **0.0280** | 0.0295 | 0.6680 |
|  | Group B | Group A | -0.2330 | 0.1282 | 0.1660 | -0.5353 | 0.0693 |
|  |  | Group C | 0.1158 | 0.1350 | 0.6670 | -0.2026 | 0.4342 |
|  | Group C | Group A | -0.3488 | 0.1353 | **0.0280** | -0.6680 | -0.0295 |
|  |  | Group B | -0.1158 | 0.1350 | 0.6670 | -0.4342 | 0.2026 |

Group A lowest tertile; Group B middle tertile; Group C highest tertile. The mean difference is significant at the 0.05 level.

**Additional Table 3.**

**Multiple comparisons between CSF HGF and** **longitudinal changes in cognitive function**

| **Dependent variable** | **CSF HGF groups** | **β** | ***p*** |
| --- | --- | --- | --- |
|  |  |  |  |
| MMSE change rate | Group B vs. Group A | -0.2565 | 0.1623 |
|  | Group C vs. Group A | -0.2155 | **0.0371** |
|  | Group C vs. Group B | -0.1478 | 0.492 |
| ADNI-MEM change rate | Group B vs. Group A | -0.0247 | 0.2788 |
|  | Group C vs. Group A | -0.0271 | **0.0397** |
|  | Group C vs. Group B | -0.0288 | 0.2720 |
| ADNI-EF change rate | Group B vs. Group A | -0.0709 | **0.0186** |
|  | Group C vs. Group A | -0.0442 | **0.0037** |
|  | Group C vs. Group B | -0.0174 | 0.5996 |

Longitudinal rates of change in cognitive function (including MMSE, ADNI-MEM , and ADNI-EF) were computed by using linear mixed models. All models were adjusted for age, sex, years of education, APOE4 status, baseline diagnosis, and baseline cognitive status. Group A lowest tertile; Group B middle tertile; Group C highest tertile. The mean difference is significant at the 0.05 level.


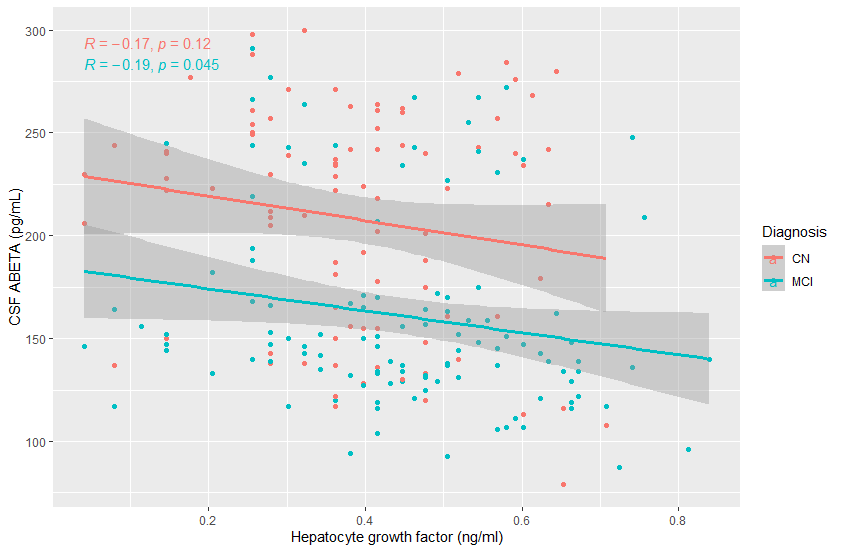


**Additional Figure 1.** Associations of baseline CSF HGF with CSF AD biomarkers in non-demented participants (stratified by diagnosis). The baseline CSF HGF is significant associated with the CSF Aβ42 in MCI (R = -0.19, P = 0.045), but not in CN (R = -0.17, P = 0.12).

**
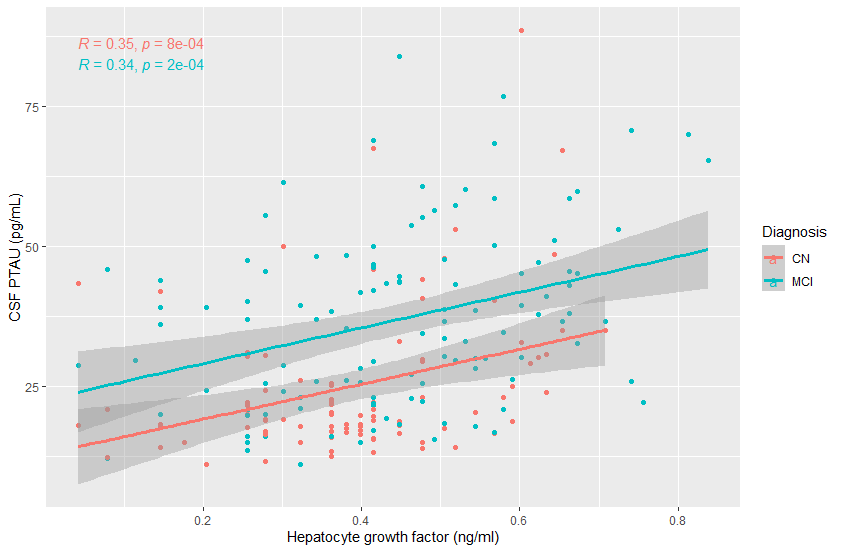
**

**Additional Figure 2.** Associations of baseline CSF HGF with CSF AD biomarkers in non-demented participants (stratified by diagnosis). The baseline CSF HGF is significant associated with the CSF pTau (CN, R = 0.35, P = 8E-04; MCI, R = 0.34, P = 2E-04).


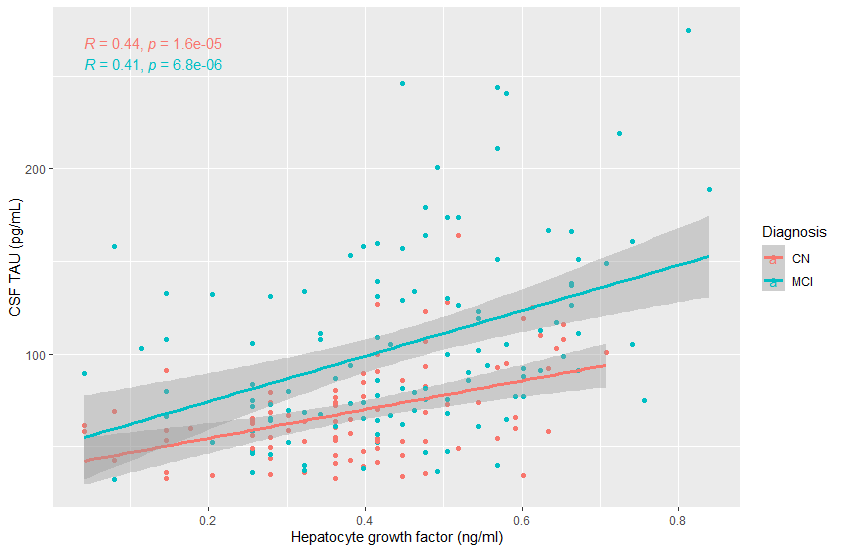


**Additional Figure 3.** Associations of baseline CSF HGF with CSF AD biomarkers in non-demented participants (stratified by diagnosis). The baseline CSF HGF is significant associated with the CSF tTau (CN, R = 0.44, P = 1.6E-05; MCI, R = 0.41, P = 6.8E-05).
